# Analysis of Melanin Structure and Biochemical Composition in Conjunctival Melanocytic Lesions Using Pump–Probe Microscopy

**DOI:** 10.1167/tvst.8.3.33

**Published:** 2019-06-04

**Authors:** Francisco E. Robles, Sanghamitra Deb, Lejla Vajzovic, Gargi K. Vora, Prithvi Mruthyunjaya, Warren S. Warren

**Affiliations:** 1 Department of Chemistry, Duke University, Durham, NC, USA; 2 Wallace H. Coulter Department of Biomedical Engineering, Georgia Institute of Technology and Emory University, Atlanta, GA, USA; 3 Beckman Institute for Advanced Science and Technology, University of Illinois at Urbana-Champaign, Urbana, IL, USA; 4 Department of Ophthalmology, Duke University Medical Center, Durham, NC, USA; 5 Department of Ophthalmology, Stanford University Medical Center, Palo Alto, CA, USA; 6 Department of Radiology, Duke University Medical Center, Durham, NC, USA; 7 Duke University, Departments of Physics, Durham, NC, USA

**Keywords:** conjunctival melanoma, primary acquired melanosis, benign conjunctival nevus, pump–probe microscopy, multiphoton microscopy

## Abstract

**Purpose:**

We analyze melanin structure and biochemical composition in conjunctival melanocytic lesions using pump-probe microscopy to assess the potential for this method to assist in melanoma diagnosis.

**Methods:**

Pump–probe microscopy interrogates transient excited-state photodynamic properties of absorbing molecules, which yields highly specific molecular information with subcellular spatial resolution. This method is applied to analyze melanin in 39 unstained, thin biopsy specimens of melanocytic conjunctival lesions. Quantitative features of the biochemical composition and structure of melanin in histopathologic specimens are assessed using a geometric representation of principal component analysis (PCA) and principles of mathematical morphology. Diagnostic power is determined using a feature selection algorithm combined with cross validation.

**Results:**

Conjunctival melanomas show higher biochemical heterogeneity and different overall biochemical composition than primary acquired melanosis of the conjunctiva (PAM) without severe atypia. The molecular signatures of PAMs with severe atypia more closely resemble melanomas than other types of PAMs. Pigment organization in the tissue becomes more disorganized as diagnosis of the lesions worsen, but nevi are more inconsistent biochemically and structurally than other lesions. Relatively high sensitivity (SE) and specificity (SP) is achieved for differentiating between various melanocytic lesions, particularly PAMs without severe atypia and melanomas (SE = 89%; SP = 87%).

**Conclusions:**

Pump–probe microscopy is a powerful tool that can identify quantitative, phenotypic differences between various types of conjunctival melanocytic lesions.

**Translational Relevance:**

This study further validates the use of pump–probe microscopy as a potential diagnostic aid for histopathologic evaluation of conjunctival melanocytic lesions.

## Introduction

Melanocytic proliferations in the conjunctiva account for more than half of all neoplasms in that anatomic region, and fall under three broad categories: melanocytic nevus, conjunctival melanosis (which includes complexion-associated melanosis and primary acquired melanosis [PAM], also called conjunctival melanocytic intraepithelial neoplasia [c-MIN]^1^), and invasive conjunctival melanoma.^2^ Invasive melanomas account for 2% of all ocular malignancies^3^ and, like cutaneous melanomas,^4^ their incidence rate is rapidly growing; for white men in the United States, for example, the indicine rate increased by 295% from 1973 to 1999.^5^ Conjunctival melanomas typically develop de novo (12%–47% of the time^6^; these also are considered the most aggressive^5^,^6^), or evolve from a subtype of conjunctival melanosis (10%–74%).^3^,^6^–^8^ Conjunctival nevi are benign proliferations that are classified similarly to cutaneous nevi and more rarely evolve to malignant melanomas (1%–26%).^6^,^8^

Accurate clinical diagnosis of a conjunctival melanocytic lesion can be challenging for a number of reasons. For example, conjunctival melanocytic proliferations contain histologic patterns that differ substantially from their cutaneous counterparts, but the pathology-based terminology used to define cutaneous melanocytic proliferations often are applied by nonocular pathologists to ocular specimens, which are not always applicable.^1^,^9^ Also, PAM is further subcategorized histopathologically by severity of atypia (no atypia, mild, moderate, or severe); while this terminology maybe useful for monitoring the progression of these lesions,^1^ the gradation is highly vulnerable to the subjective nature of histopathologic interpretation. Plus, there often exists an overlap in appearance of PAM and relatively flat or diffuse configurations of conjunctival melanoma. In fact, some experts argue that PAMs with severe atypia should be considered melanomas in situ^7^,^9^,^10^ – the vast majority of PAMs that evolve to invasive melanomas fall under this subcategory^7^,^9^ and should be treated as such. Moreover, the most recent update of the American Joint Committee on Cancer clinical classification describes the T(is) stage as conjunctival melanoma in situ, or melanoma confined to the epithelium. This condition includes primary acquired melanosis with atypia in more than 75% of normal epithelial thickness.^11^ These considerations complicate reliable and reproducible clinical and histopathologic evaluation.

Noninvasive imaging technologies have the potential to aid in the diagnosis of these melanocytic lesions, but to date such imaging interventions have been limited. For example, confocal microscopy,^12^ optical coherence tomography,^13^ and ultrasound^14^ have been used to provide three-dimensional (3D), tomographic images of the structure of conjunctival lesions; but the source of contrast for these methods, optical or acoustic scattering, fails to provide direct disease-specific information that can help assess the pathology. Thus, their use has not been widely adopted (but continue to be studied). To alleviate these shortcomings, we have developed pump–probe microscopy, a novel label-free molecular imaging method that provides unique insight into the biochemical composition of melanin, which serves as an indicator of melanocyte activity that can be used as a biomarker of disease.^15^–^17^

In short, pump–probe microscopy applies a two-color pulsed laser system to interrogate the ultrafast transient excited and ground state photodynamic properties of pigments. Implementation of this approach is related to laser scanning microscopy, but yields highly specific biochemical information without exogenous agents. We have shown that pump–probe microscopy is sensitive not just to the type of melanin (eu- and pheo-melanin), but also oxidation state, aggregate size, and metal content.^18^ Using thin, unstained biopsy specimens, we leveraged this biochemical sensitivity to quantitatively differentiate melanomas from melanocytic nevi and to provide novel insight into the metastatic potential of invasive melanomas in the skin^15^,^16^,^19^ and female genital tract.^17^ Using a small sample set (*N* = 8), we also showed that pump–probe microscopy can identify average differences in pigmentation patterns among conjunctival nevi, PAMs, and melanomas, and that pigment in the conjunctiva is not identical to that found in cutaneous lesions.^20^

In this work, we significantly increased our sample set (*N* = 39) to better evaluate the diagnostic use of pump–probe microscopy for melanocytic proliferations in the conjunctiva. We applied a geometric representation of principal component analysis (PCA) and used principles of mathematical morphology to quantify the molecular signatures alongside the spatial structure of the pigment. A robust statistical analysis was applied to assess the ability of pump–probe microscopy to classify conjunctival melanocytic lesions. Results demonstrated that this novel molecular imaging method is a promising tool for differentiating various types of conjunctival lesions, particularly PAMs without severe atypia and melanomas. Data also indicated that PAMs with severe atypia share a close pigment phenotypic relationship with melanomas, providing some additional support to the notion that the former lesions may be better categorized as melanomas in-situ.

## Methods

We analyzed 39 previously excised conjunctival melanocytic lesions (Table 1). From each fixed tissue specimen, two adjacent, thin (∼10 μm) sections were cut, where one was left unstained while the other was stained with hematoxylin and eosin (HE). An ophthalmic pathology expert used the HE-stained sections to classify regions of interest; then, these regions were identified in the adjacent, unstained sections and imaged with pump–probe microscopy. A total of 471 pump–probe images were acquired. These studies were conducted under a protocol approved by the Duke University School of Medicine institutional review board.

**Table 1:** Sample Set Characteristics

	No. of Images^a^	Sections with Tissue Type	Patient Diagnosis
Nevi	145	16	16
PAM w/o atypia and w/ mild/mod atypia	85	10	9
PAM w/ severe atypia	47	4	3
Melanoma	194	10	11
Total	471	40	39

Using PAM with severe atypia as an example, the sample set contained three specimens from patients diagnosed with PAM with severe atypia, but four total sections in the study contained indications of PAM with severe atypia. The extra section with this tissue type came from a patient with melanoma whose tissue has regions more consistent with PAM with severe atypia. A total of 47 images were taken from regions with PAM with severe atypia. w/o, without; w/, with; mod, moderate.

a Each image region is a 360 × 360 μm^2^ area.

The pump–probe system has been described in detail previously.^15^,^21^,^22^ Briefly, we used a custom-built laser scanning microscope, equipped with a dual-output (i.e., two color) femtosecond laser system. The two outputs were derived from a Coherent Mira optical parametric oscillator (OPO; Coherent, Inc., Santa Clara, CA) and a 80 MHz Chameleon Ti:Sapphire oscillator (Coherent, Inc.), tuned to 730 and 810 nm, respectively serving as the pump and probe pulses. The laser pulses used less than 0.65 mW total power to avoid any effects that may alter the melanin pigment chemistry (e.g., photobleaching).^18^

**Figure 1:**
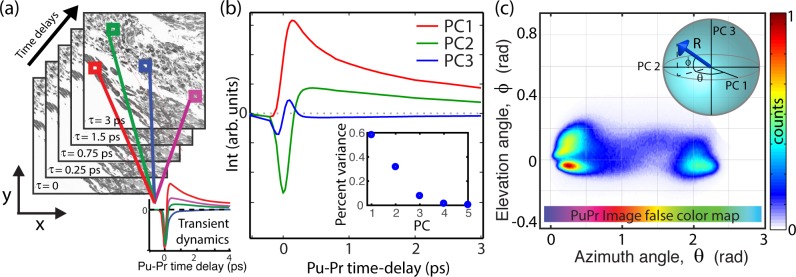
(a) Illustration of pump–probe microscopy data. Data acquired to generate pump–probe images are 3D, with two spatial dimensions (x and y) and the third consisting of the transient photodynamics. (b) Transient signals from melanin are processed with principle component analysis, where the top three principal components (pc's) capture over 97% of the data variance. (c) 2D histogram of melanin transient signals. The top three pc's span a 3D space, whose angles (in spherical coordinates) can be used to graphically represent the biochemical composition, as shown in the inset.

Molecular signatures were obtained by first modulating the 730-nm pump beam at 2 MHz, while leaving the 810-nm probe beam unmodulated. Both beams were spatially overlapped and then sent collinearly to the microscope. When the beams were focused onto the sample, nonlinear optical interactions between the pump and probe (which interrogate electronic excited and ground states of the sample) transferred the 2-MHz modulation to the probe. This signal then was detected with a photodiode and a lock-in amplifier. Finally, the ultrafast photodynamic properties were obtained by varying the time-delay between the pump and probe pulses. A total of 34 time-delays, ranging from approximately 0 to 4 picoseconds (ps), were used to determine the photodynamic properties. The data acquired were 3D, with two dimensions being the *x* and *y* coordinates of the thin sample, and the third being the pump–probe time-delays, which yielded the ultrafast transient dynamics (i.e., molecular signatures; Fig. 1). Each individual pump–probe image had a field of view of 360 × 360 μm.

To quantify the molecular signatures, we used a geometric representation of principal component (PC) analysis.^16^,^23^,^24^ This procedure (Fig. 1) allowed us to reduce the dimensionality of the data set (from 34 time-delays to only a few PCs) for quantitative analysis and enabled a visual representation of the signals. Here, we leveraged the fact that >97% of the signal variance could be described by the top 3 PCs. This could be represented in a spherical coordinate system, where the angles (*θ* and *φ*) describe the transient dynamics, while the radius (R) represents the signal strength (i.e., relative concentration). For image display, we used a hue saturation value color map scheme, where the hue was set based on *θ* (Fig. 1c, inset), the value is set by R, and the saturation is set to 1.

## Results

### Pump–Probe Microscopy Images of Melanocytic Proliferations in the Conjunctiva

Pump–probe signals arising from melanin (excluding surgical ink and hemoglobin) from the entire sample set were first processed with PCA to derive the top PCs. As the inset of Figure 1b shows, the top 3 PCs correspond to >97% of the variance, which then were used to construct a visual representation of the pump–probe signals (Fig. 1c) based on the angles of the PCs spherical coordinate system, as described above. As Figure 1c shows, the two-dimensional (2D) histogram of the entire data set reveals two major components (also known as endmembers),^16^,^23^ with all other signals comprising a linear combination of these two components. One endmember is located at an azimuth angle of *θ* = 0 rad (i.e., a positive contribution from PC1), which signifies a loss of nonlinear interaction (e.g., excited state absorption). These types of signals have been observed in melanins with large aggregates and those void of metals.^18^ The other endmember lies at approximately *θ* = 2.25 rad (negative contribution from PC1, and a positive contribution from PC2) and indicates a gain in the probe from interactions, like ground-state bleaching. These signals have been observed in melanins with high levels of metal content, small aggregates, and synthetic pheomelanin.^18^,^25^ Thus, the pump–probe signals, and hence the mapping of the signals onto this principal component space, gives an indication of melanin biochemical composition (albeit not uniquely). A more detailed analysis on how the pump–probe signals differ based on chemical composition has been reported previously.^18^,^25^ The mapping of each spatial pixel's molecular signature on the *θ*-axis also defines the color assignment in the pump–probe images – the color map is illustrated in Figure 1c (horizontal color map).

**Figure 2:**
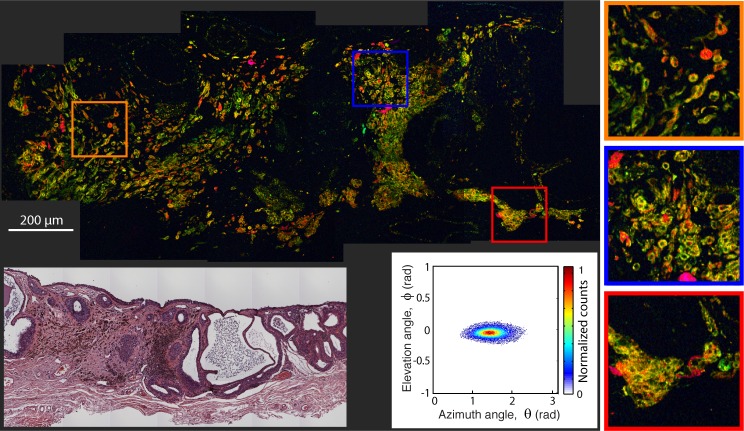
Pump–probe microcopy image of unstained benign nevus biopsy sample, along with its 2D histogram of the pc's angular distribution and adjacent, HE-stained microphotograph.

**Figure 3:**
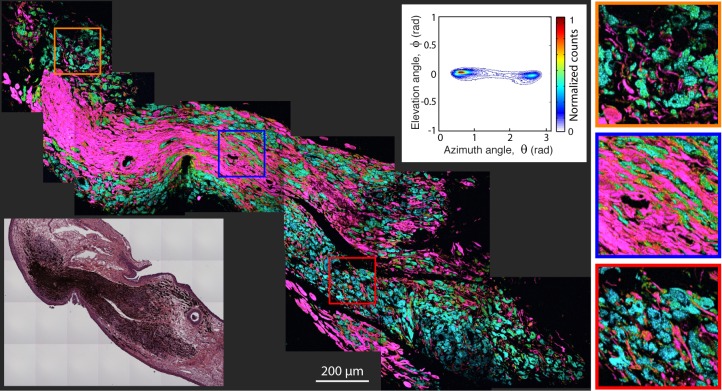
Pump–probe microcopy image of unstained benign blue nevus biopsy sample, along with its 2D histogram of the pc's angular distribution and adjacent, HE-stained microphotograph.

**Figure 4:**
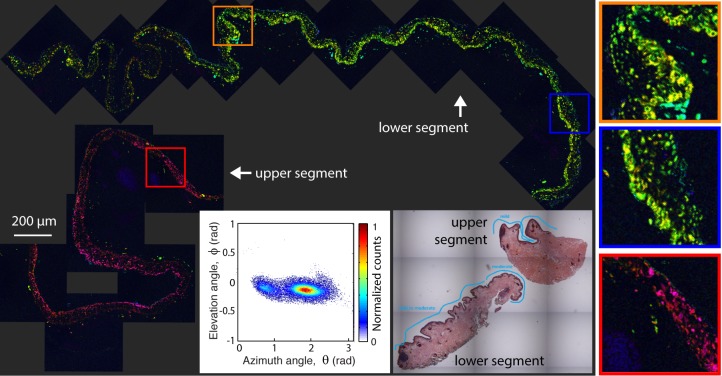
Pump–probe microcopy image of PAM with mild-to-moderate atypia, along with its 2D histogram of the pc's angular distribution and adjacent, HE-stained microphotograph.

**Figure 5:**
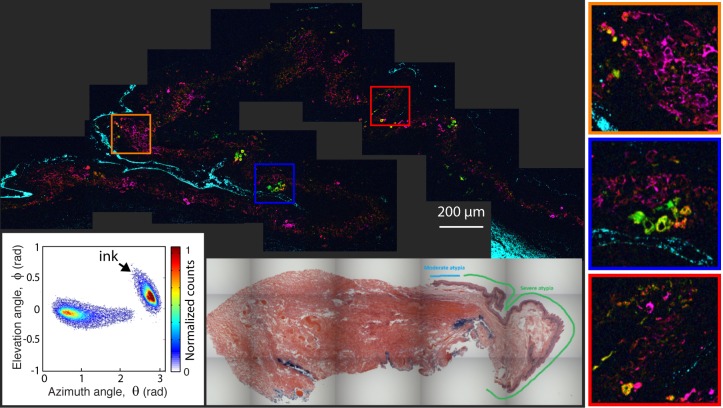
Pump–probe microcopy image of PAM with severe atypia, along with its 2D histogram of the pc's angular distribution and adjacent, HE-stained microphotograph.

**Figure 6:**
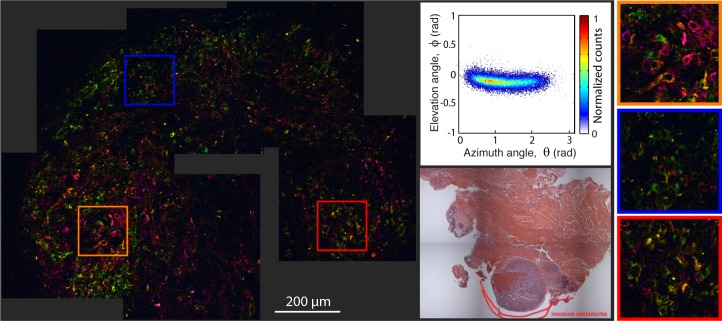
Pump–probe microcopy image of invasive conjunctival melanoma, along with its 2D histogram of the pc's angular distribution and adjacent, HE-stained microphotograph.

Figures 2 to 6 illustrate selected pump–probe images of unstained tissue sections, along with their 2D histogram of the PCs angular distribution and the adjacent H&E stained sections. Figure 2 shows a benign nevus with representative characteristics of these lesions in our sample set. However, as we discuss below, these lesions show a wide spectrum of features, which is a hallmark of nevi.^9^,^11^ This lesion is highly pigmented with a moderate level of biochemical heterogeneity, as represented by the different colors in the pump–probe image and the spread of the signals in the 2D histogram. The pigment is primarily in the substantia propria, with a high degree of structural heterogeneity, meaning that the pigment is not well organized or confined to a particular area. Figure 3 illustrated a different type of benign nevus, a blue nevus. This unique lesion shows a biochemical composition unlike any other investigated to date, with a strongly bimodal distribution. One cluster, which appears to correspond to spindle melanocytes or melanin pigment around the fibrotic stroma, shows a distinct pink hue from pump–probe signals that closely resemble the first pc (signifying a loss nonlinear interaction indicative of large melanin aggregates and melanins void of metals). The other cluster, in cyan, has the opposite pump–probe response – mostly a negative contribution from the first PC, signifying a gain in the probe pulse (resulting from melanin with high levels of metal content, small aggregate size, or pheomelanin). The morphology suggests that this response is from melanin in the cytoplasm of plump, epithelioid melanocytes, which are characteristic of this type of lesion.^10^,^26^

Next, we showed two PAMs. The first (Fig. 4) is a PAM with mild-to-moderate atypia, with the tissue section containing two separate segments (from the same patient) that exhibit slightly different biochemical characteristics. Note that while the color of each segment appears drastically different, in fact the molecular signatures are only slightly different as seen by the small separation of the two clusters (one from each segment) on the same 2D histogram. In both pieces, the pigment is found diffusely through the epithelium with relatively elevated concentrations in the basal layer. Overall, the lesion shows a higher degree of organized pigment structure compared to the nevi, meaning that the pigment is confined to a particular region and it resembles the structure of normal tissue. The faint dark blue circles in the upper segment are from hemoglobin, which have *θ* ≲ 0. The second PAM (Fig. 5) contains severe atypia, and exhibits a more disorganized and widespread pigment structure, with a few scattered cells possessing different pump–probe (and, thus, molecular) signatures compared to the majority of the surrounding pigment. In this image, the cyan color corresponds to surgical ink. Note that the elevation angle, *ϕ*, is different for the ink than the melanin seen in the blue nevus even though both are mapped to a cyan hue.

Finally, Figure 6 shows an invasive melanoma. Here, the pigment is highly heterogeneous, structurally and biochemically. The pigment is in the epithelium and substantia propria, and has a finer texture compared to the coarser pigment structures found in the nevi and PAMs without severe atypia. Pigment in the cytoplasm also reveals many cells with large nucleus and high nucleus-to-cytoplasm ratio.

### Image Quantification and Multivariate Analysis

To quantify different features of the conjunctival lesions, we used parameters derived from the melanin biochemical composition and its structure. Average parameters of the biochemical composition of the lesions (i.e., that do not take structure into account) are readily assessed using the 2D PC histograms, where we extract the mean, standard deviation, and entropy of the distribution. Then, to account for the overall structure and structure of each endmember, we use mathematical morphology.^16^,^19^ In this process, three gray scale images were produced. The first consisted of the total melanin pigment obtained from the magnitude of the signal (R in Fig. 1c), which suppresses the detailed biochemical information. The other two gray-scaled imaged are weighted by the relative concentration of the two endmembers. For one, the R image is multiplied by 1 for pixels with *θ* = 2.5 and 0 for pixels with *θ* = 0 rad (denoted *θ*+), and the other uses the opposite scale (denoted *θ*−). These two images capture the structural and biochemical information of the melanin. Finally, we applied a 2D mathematical autocorrelation transformation and extracted second-order geometric information (i.e., morphologic covariance) to quantify the structure. Details of this method have been reported previously.^19^ Overall, 30 features are extracted from each pump–probe image.

Finally, we applied a multivariate analysis consisting of a feature selection algorithm to identify the features with the most diagnostic value, followed by cross-validation. Details for this method have been described previously by Robles et al.^16^ In short, forward sequential feature selection was used in a wrapper fashion with data drawn from individual images to capture the most amount of variation across the data. Once features were selected, we assessed the diagnostic power (sensitivity [SE] and specificity [SP]) by applying the leave-one-out cross validation (LOOCV) method, with all images from one patient treated as one ‘sample' so that they are withheld from training to ensure that the training set was independent of the test set. While LOOCV does have its limitations, including possible bias due to a priori knowledge of the diagnosis, it is a well-accepted method to obtain some measure of predictive power when the sample size is small.

**Figure 7:**
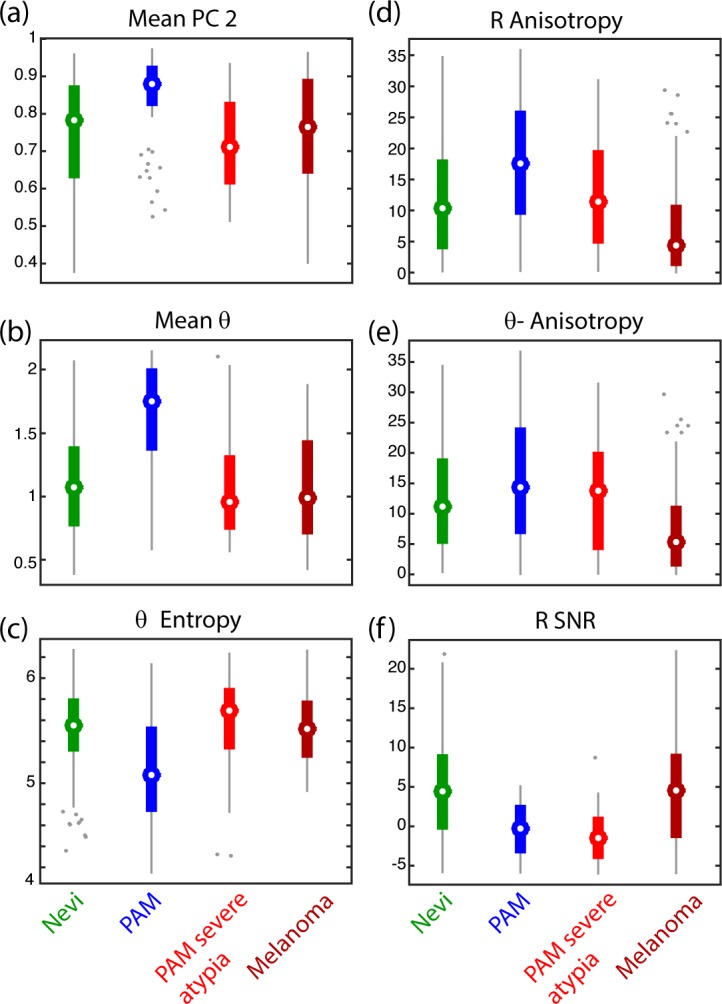
(a–f) Box plots summarizing selected features used to differentiate between various conjunctival melanocytic lesions. Features on the left column (a–c) only take the biochemical composition into account, while features on the right column (d–f) are derived from the structure and biochemical composition of melanin.

Figure 7 shows the most important features that distinguish nevi, PAMs without severe atypia, PAMs with severe atypia, and melanomas – the features correspond to a combination of average biochemical composition (left column) as well as the pigment structure (right column). The data present several interesting trends, starting with the apparent unique biochemical composition of PAMs without severe atypia. Here, they show a higher *θ*-value, signifying a more negative pump–probe response compared to melanomas. This also is reflected by a higher value of the second PC. Both results are consistent with our previous finding in the smaller pilot study.^20^ The lower *θ* entropy for PAMs compared to melanomas suggests a lower biochemical heterogeneity, which is consistent with the observations provided above. Interestingly, PAMs with severe atypia exhibit an average pigment composition that more closely resembles melanomas instead of PAMs without severe atypia. Nevi also show a very wide and heterogeneous biochemical composition, consistent with our previous observations.

The structural analysis also revealed an interesting progression from PAMs without severe atypia to melanoma. Specifically, PAMs without severe atypia have the highest level of anisotropy in the R and θ images, which describes its well-bounded pigment in the epithelium. As the atypia worsens and then progresses to melanoma, the pigment spreads and results in lower levels of anisotropy. Similar trends have been observed for cutaneous and vulvar pigmented lesions.^17^ The nevi, however, did not follow this trend, and again showed much higher variability in their pigment structure. The last structural parameter, R signal-to-noise ratio (SNR), is a measure of the overall level of pigmentation (ignoring biochemical composition), and showed that melanomas and nevi express the most pigment.

Table 2 presents the LOOCV results. PAMs without severe atypia can be differentiated from melanomas with high SE (89%) and SP (87%). However, it remains difficult to separate the PAMs with severe atypia from either melanoma (SE = 87%, SP = 57%) and from PAMs without severe atypia (SE = 55%, SP = 85%). This problem likely is due to the low number of lesions available with PAMs with severe atypia, but also is compounded by the subtle continuum that differentiates these lesions. A larger sample set could reveal a more robust set of features that help assess quantitative differences between these subgroups. Separation of the nevi from PAMs without severe atypia is fairly robust, but degrades slightly for PAMs with severe atypia and melanomas.

**Table 2:** Sensitivity and Specificity as Determined by LOOCV

	SE, %	SP, %	No. Lesions
PAM w/o atypia and w/ mild/mod atypia vs. melanoma	89	87	20
PAM w/ severe atypia vs. melanoma	87	57	15
PAM w/o atypia and w/ mild/mod atypia vs. PAM w/ severe atypia	55	85	12
Nevi vs. melanoma	72	64	27
Nevi vs. PAM w/o atypia and w/ mild/mod atypia	86	83	25
Nevi vs. PAM w/ severe atypia	70	76	20

## Discussion

We have shown that pump–probe microscopy is a powerful tool with the potential to aid in the diagnosis of conjunctival melanocytic lesions. Results showed that melanomas have higher biochemical heterogeneity and different average biochemical composition than PAMs without severe atypia. The molecular signatures of PAMs with severe atypia, on the other hand, more closely resemble melanomas than other types of PAMs, in support of the notion that these lesions may be better categorized as melanomas in-situ. Structurally, the pigment becomes more disorganized as diagnosis of the lesions worsened. Data also showed that nevi have a more inconsistent biochemical and structural composition than other lesions. Using six (of 30) parameters that quantify the structure along with the biochemical composition of the melanin pigment, we demonstrated a relatively high sensitivity and specificity for differentiating between various melanocytic lesions, particularly PAMs without severe atypia and melanomas.

This study focused on excised, thin tissue biopsy specimens, and as such we have shown that pump–probe microscopy can serve as a quantitative tool to achieve a more accurate diagnosis, one rooted on an endogenous molecular phenotype (i.e., melanin expression) that indicates malignancy. While this study used a moderate sample size for conjunctival melanocytic lesions, a larger and, more importantly, prospective study will be needed to more definitively determine the diagnostic use of pump–probe microscopy.

It also is important to note that while the processing and/or fixing protocols of these samples may differ, the pump–probe signals from melanin are remarkably stable. Data (not shown) indicated that the pump–probe signals (from human brown hair) remain invariant to different duration of fixation and materials. These results are further supported by the fact that all of the (hundreds of) samples we tested (from skin, conjunctiva, and so forth), which have surely not been processed in the exact same way, possess the same general set of characteristics. This is not too surprising given that the pump–probe signals come from melanin, not associated with proteins that are crosslinked in the fixation process; thus, the fixation has much less effect on the melanin pump–probe signals. We also showed that the pump−probe signature of eumelanin from the ink sac of a Jurassic cephalopod is identical to that of its modern-day counterpart,^27^ further validating the stability of melanin, and pump–probe signals, for analyzing archived pigmented tissue.

Finally, in vivo imaging with pump–probe microscopy is possible,^28^ albeit with more limited access to the biochemical composition,^29^ and in the future it may provide similar information without the need for excisional biopsy. Our study not only will serve as a roadmap to future endeavors for in vivo imaging, but it also stands alone by establishing pump–probe microscopy as a potential diagnostic aid for histopathologic evaluation of conjunctival melanocytic lesions.
